# The first report on complete mitochondrial genome of *Murina yushuensis* (Chiroptera: Vespertilionidae) and its phylogenetic analysis

**DOI:** 10.1080/23802359.2025.2604863

**Published:** 2025-12-24

**Authors:** Hongyan Shi, Taihan Huang, Xindan Fan, Xiaoyun Wang, Wenhua Yu, Xiaoxue Fu, Yi Wu

**Affiliations:** ^a^ Ecological Security and Protection Key Laboratory of Sichuan Province, Key Laboratory of Research and Conservation of Biological Diversity in Minshan Mountain of National Park of Giant Pandas, College of Life Sciences, Mianyang Normal University, Mianyang, China; ^b^ Key Laboratory of Southwest China Wildlife Resources Conservation, China West Normal University, Ministry of Education, Nanchong, China; ^c^ South China Biodiversity Research Center, School of Life Sciences, Guangzhou University, Guangzhou, China

**Keywords:** Mitogenome, Yushu tube-nosed bat, phylogeny analysis

## Abstract

In this study, a complete mitochondrial genome of *Murina yushuensis,* sampled from Sichuan, China, was sequenced using the Illumina NovaSeq 6000 S4 platform. Results showed that the mitochondrial genome was a circular structure with a total length of 16,520 bp, comprising 13 protein-coding genes, 22 *tRNA* genes, two *rRNA* genes and 1 control region. Phylogenetic analysis based on 13 protein coding genes from limited mitochondrial genome data of 10 species of *Murina* revealed that *M. yushuensis* is closely related to *M. eleryi*. This study provides genetic data for further taxonomic and phylogenetic researches on *Murina* bats.

## Introduction

*Murina yushuensis* Yu et al. (2020) (Vespertilionidae, Chiroptera) was first described by Wang et al. (2024a) based on a male tube-nosed bat collected from an arid cave located in Yushu City, Qinghai Province, China. This small-sized forest bat is characterized by its black-based brown-gold dorsal fur and black-based grayish ventral fur. Subsequently, Shi et al. (2025) also found its distribution in Sichuan Province and Xizang Autonomous Region of China, based on phylogenetic evidence from mitochondrial cytochrome c oxidase subunit I (*COI*). So far, the species is only found in the above mentioned three locations. Given that *M. yushuensis* is a recently discribed species with a limited number of specimens, research on it is relatively scarce, and there are no reports on its complete mitochondrial genome, except for one *COI* sequence from the type specimen. Therefore, this study sequenced, assembled and annotated the complete mitochondrial genome of *M. yushuensis*, which is expected to provide basic data for the classification and phylogenetic analysis of this species.

## Materials and methods

**Figure 1.**
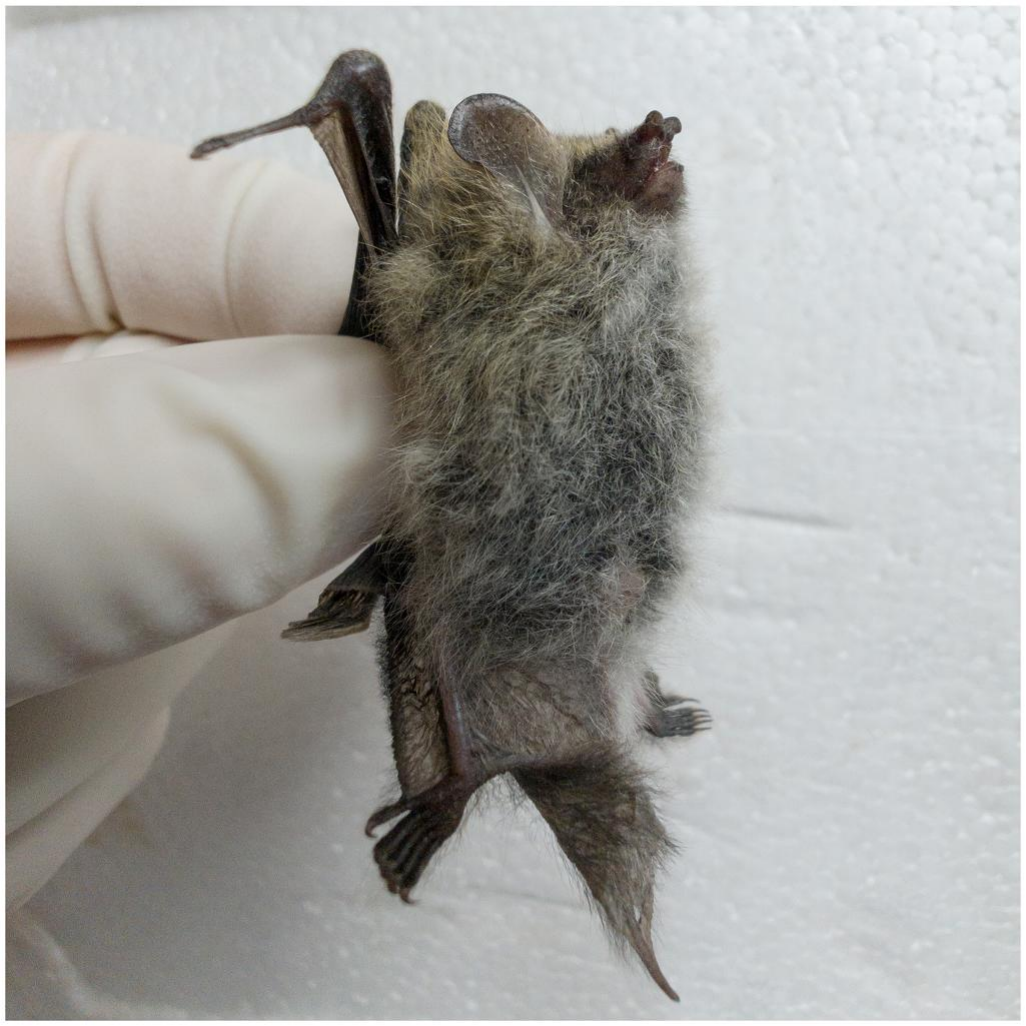
*Murina yushuensis* specimen (MYTC24172) collected from Huanglong Nature Reserve in Songpan County, Sichuan Province of China (the photo was taken by Taihan Huang).

The sample individual (voucher number MYTC24172) (Figure 1) was collected in a forest trail at an elevation of 2,929 m within Huanglong Nature Reserve in Songpan County, Sichuan Province of China (32.81102252°N, 103.92003352°E), in September 2024, using a two-bank harp trap. The bat has a small body size, with a body mass of 4.41 g and a forearm length of 31.09 mm.The dorsal pelage has dark golden-brown tips with black bases, while the ventral fur has whitish-gray tips tinged with sandy yellow and black bases. This species can be distinguished from similar species (*M. aurata, M. chrysochaetes, M. harpioloides, M. yuanyang*) by its slightly duller fur coloration. Following euthanasia *via* inhalation of excessive carbon dioxide, chest muscle tissue was excised and stored in an ultra-low-temperature freezer at −80 °C. The specimen is currently preserved in the Animal Collection of Mianyang Normal University (Hongyan Shi, Contact: shylh310@163.com). Total genomic DNA was extracted from 20 mg of chest muscle tissue using the TIANamp Genomic DNA Kit (TIANGEN, Beijing, China), followed by paired-end sequencing (150 bp) on an Illumina Novoseq 6000 S4 platform, which generated approximately 30 G of raw sequencing data. Mitochondrial genomes were assembled and annotated using MitoZ v3.6 (Meng et al. 2019) with 2 G bp of raw fastq data (The coverage-depth map was shown in Figure S1). The mitochondrial genome map was drawn on the OGDRAW online platform (https://chlorobox.mpimp-golm.mpg.de/OGDraw.html).

We performed maximum likelihood (ML) analyses using 13 protein-coding genes (PCGs) with third codon positions removed. Mitochondrial genome sequences of *Harpiocephalus harpia*, which was selected as outgroup, and other nine species within the genus *Murina* were downloaded from NCBI database. The 13 PCGs were extracted using PhyloSuite v1.2.3 (Zhang et al. 2020) and aligned with MAFFT v7.505 (Katoh and Standley 2013). The alignments were codon-adjusted using MACSE v2.0.6 (Ranwez et al. 2018) to minimize frame-shift errors. Low-quality alignment regions were filtered with Gblocks v0.91b (Talavera and Castresana 2007) under default parameters. After removing the third codon site, the trimmed sequences of the 13 PCGs were concatenated, and the optimal partitioning scheme along with substitution models were selected *via* ModelFinder v2.2.0 (Kalyaanamoorthy et al. 2017) (Table S1). Maximum likelihood (ML) phylogenetic trees were reconstructed using IQ-Tree v2.4.0 (Nguyen et al. 2015) with nodal support assessed through 5,000 ultrafast bootstrap (UFboot) replicates and 1,000 Shimodaira-Hasegawa approximate likelihood ratio test (SH-aLRT) replicates. Branches were considered strongly supported if they met the thresholds of SH-aLRT ≥80% and UFboot ≥95%.

## Results

**Figure 2.**
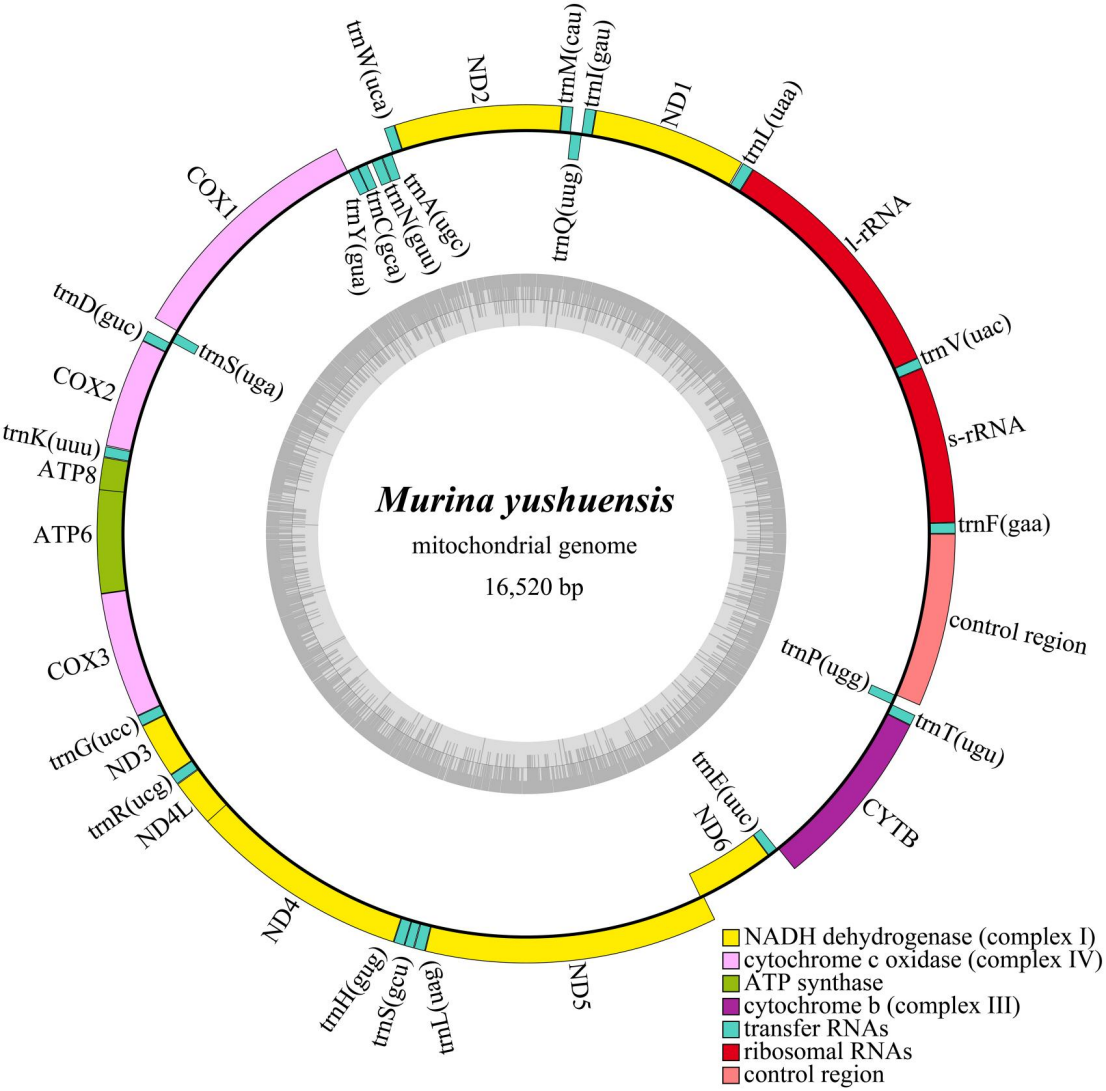
Mitochondrial genome map of *Murina yushuensis*. Different colors represent genes with different functions.

**Figure 3.**
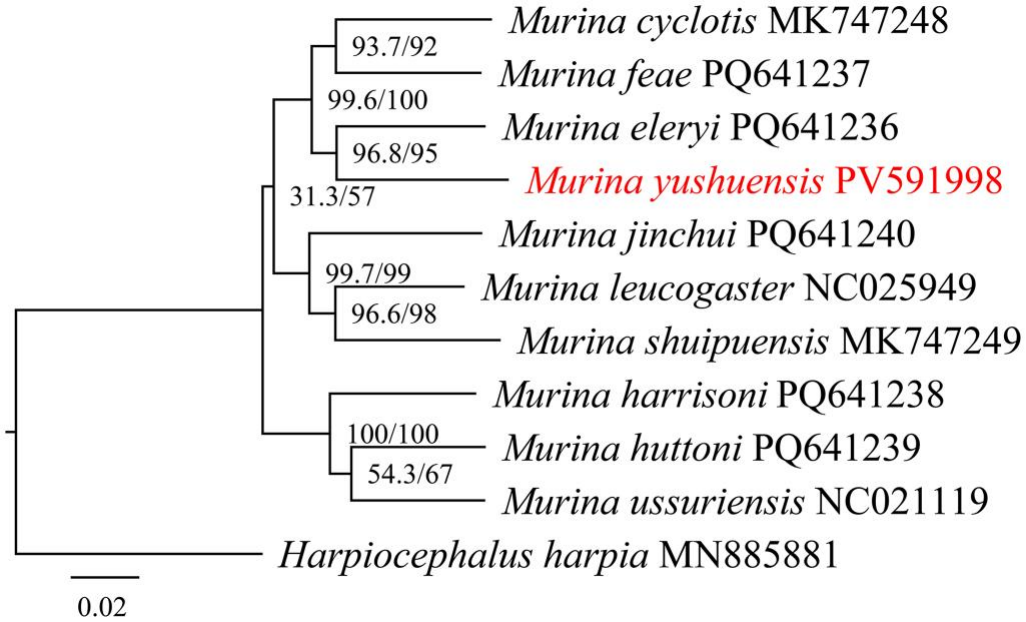
A maximum likelihood tree based on 13PCGs of genus *Murina*. The numbers shown between branches indicate the SH-aLRT (left) and UFboot (right) respectively. The following sequences were used: *Murina cyclotis* MK747248 (Yue et al. 2019), *Murina eleryi* PQ641236 (Wang et al. 2024b), *Murina feae* PQ641237 (Wang et al. 2024b), *Murina harrisoni* PQ641238 (Wang et al. 2024b), *Murina huttoni* PQ641239 (Wang et al. 2024b), *Murina jinchui* PQ641240 (Wang et al. 2024b), *Murina leucogaster* NC025949 (Yoon and Park 2016), *Murina shuipuensis* MK747249 (Huang et al. 2019), *Murina ussuriensis* NC021119 (Yoon et al. 2013), *Murina yushuensis* PV591998 (this study), *Harpiocephalus harpia* MN885881 (Tang et al. 2020).

The complete mitochondrial genome of *M. yushuensis* is 16,520 bp in length, forming a closed circular double-stranded structure, with 13 PCGs, 22 transfer RNA (*tRNA*) genes, two ribosomal RNA (*rRNA*), and one control region (*D-loop*) (Figure 2). The nucleotide composition of the entire mitogenome was 29.76% for thymine (T), 23.18% for cytosine (C), 13.33% for guanine (G), 33.73% for adenine (A). Similar to other *Murina* bats, the G + C content was 36.51%, which was lower than A + T nucleotide bias. In this genome, eight *tRNA* genes and the *ND6* gene are encoded on the light strand, whereas the other genes are located on the heavy strand. The start codons of 13 PCGs were all ATG except *ND2* (ATA), *ND3* (ATT) and *ND5* (ATA). In the termination codon, *CYTB* was AGA, five genes were incomplete codon T- - (*ND2*, *COX3*, *ND4*) or TA- (*ND1*, *ND3*), and the remaining seven genes were TAA. The phylogenetic analysis reveals that *M. yushuensis* is closely related to *M. eleryi* (Figure 3).

## Discussion and conclusion

Although 43 species of *Murina* bats have been identified worldwide (Wilson and Mittermeier 2019; Yu et al. 2020; Mou et al. 2024; Wang et al. 2024a; Mou et al. 2025), mitochondrial genome data was still scarce, with only nine available. Herein, the mitochondrial genome of *M. yushuensis* is presented for the first time. Comparative analysis revealed that the mitochondrial genome of *M. yushuensis* exhibits high structural conservation with other *Murina* bats (Yoon et al. 2013; Yoon and Park 2016; Huang et al. 2019; Yue et al. 2019). Maximum likelihood phylogenetic reconstruction based on 13 PCGs revealed the closest phylogenetic relationship between *M. yushuensis* and *M. eleryi* among the examined species. However, due to limited availability of mitochondrial genome for *Murina*, comprehensive phylogenetic inference within this genus remains constrained. The phylogenetic development based on *COI* gene by Wang et al. (2024a) showed that *M. yushuensis* was closely related to *M. harpioloides* and *M. chrysochaetes*. Consequently, expanded mitochondrial genomic datasets from *Murina* specimens are required to clarify the persistent taxonomic ambiguities within this group.

## Supplementary Material

Supplementary Material.docx

## Data Availability

The genome sequence data supporting this study are openly available in GenBank of NCBI at https://www.ncbi.nlm.nih.gov under the accession number PV591998. The associated BioProject, SRA, and Biosample numbers are PRJNA1280258, SRR34089965, and SAMN49518725, respectively.
