# Supplementary material for: The first report on complete mitochondrial genome of Murina yushuensis (Chiroptera: Vespertilionidae) and its phylogenetic analysis
Source: Mitochondrial DNA B Resour. 2025 Dec 24;11(1):171–4. doi: 10.1080/23802359.2025.2604863 (PMC12777756; doi:10.1080/23802359.2025.2604863)
Supplement: Supplementary Material.docx [file TMDN_A_2604863_SM9796.docx]

**Supplementary materials**

**The first complete mitochondrial genome of *Murina yushuensis* (Chiroptera: Vespertilionidae) and phylogenetic analysis**

Hongyan Shi^a*^, Taihan Huang^ab*^, Xindan Fan ^c^, Xiaoyun Wang^c^ , Wenhua Yu ^c^, Xiaoxue Fu^a^ & Yi Wu^c^

^a^ Ecological Security and Protection Key Laboratory of Sichuan Province，Key laboratory of research and conservation of biological diversity in Minshan mountain of national park of giant pandas, College of Life Sciences， Mianyang Normal University, Mianyang, China; ^b^Key Laboratory of Southwest China Wildlife Resources Conservation, China West Normal University, Ministry of Education, Nanchong, China; ^c^Key Laboratory of Conservation and Application in Biodiversity of South China, School of Life Sciences, Guangzhou University, Guangzhou, China

CONTACT: [wuyi@gzhu.edu.cn](mailto:wuyi@gzhu.edu.cn); [shylh310@163.com](mailto:shylh310@163.com)

^*^Co-first authors


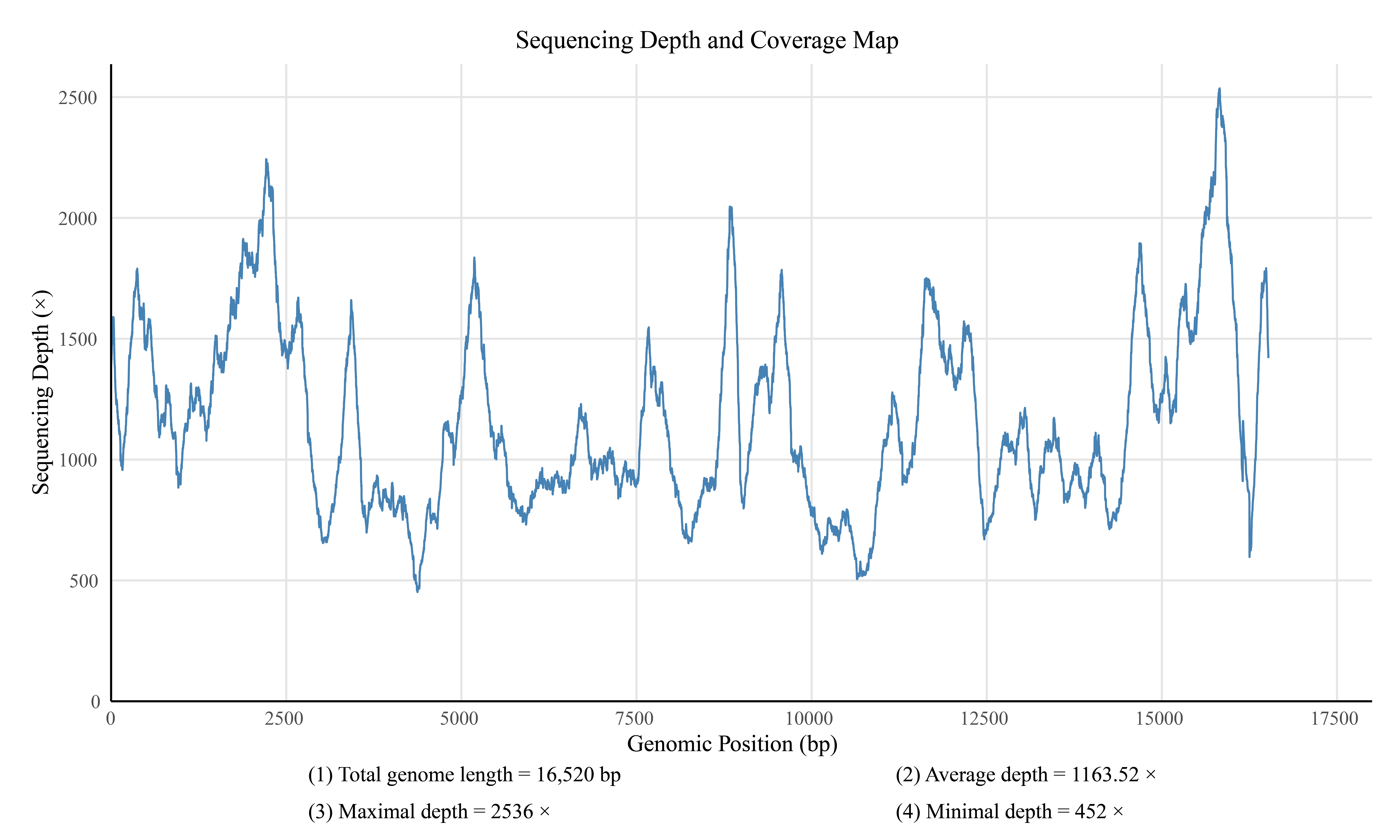


**Figure S1.** Sequencing depth and coverage map of the *Murina yushuensis* Mitochondrial genome.

**Table S1**. The partition schemes and substitution models used for phylogenetic analyses of the mitochondrial PCGs dataset.

| Subset partitions | Best model |
| --- | --- |
| P1: (atp6_codonA, atp8_codonB, cox2_codonA, cox3_codonA, cytb_codonA) | TIM2+F+G4 |
| P2: (atp6_codonB, cox1_codonB, cox2_codonB, cox3_codonB, cytb_codonB, nad1_codonB) | HKY+F+R2 |
| P3: (atp8_codonA, nad2_codonA, nad3_codonA, nad4L_codonA) | TIM3+F+R2 |
| P4: (cox1_codonA) | TNe+I |
| P5: (nad1_codonA, nad4_codonA, nad5_codonA) | TIM2+F+G4 |
| P6: (nad2_codonB, nad3_codonB, nad4L_codonB, nad4_codonB, nad5_codonB, nad6_codonB) | TPM3u+F+R2 |
| P7: (nad6_codonA) | TN+F+G4 |
